# SARS-CoV-2 ORF8 drives osteoclastogenesis in preexisting immune-mediated inflammatory diseases

**DOI:** 10.1172/jci.insight.178820

**Published:** 2024-12-20

**Authors:** Ivonne Melano, Tamiris Azamor, Camila C.S. Caetano, Nikki M. Meyer, Chineme Onwubueke, Anabelle Visperas, Débora Familiar-Macedo, Gielenny M. Salem, Brandy-Lee Soos, Cassandra M. Calabrese, Youn Jung Choi, Shuyang Chen, Younho Choi, Xianfang Wu, Zilton Vasconcelos, Suzy A.A. Comhair, Karin Nielsen-Saines, Leonard H. Calabrese, M. Elaine Husni, Jae U. Jung, Nicolas S. Piuzzi, Suan-Sin Foo, Weiqiang Chen

**Affiliations:** 1 Infection Biology Program, Global Center for Pathogen Research and Human Health, Lerner Research Institute, Cleveland Clinic, Cleveland, Ohio, USA.; 2 Fundação Oswaldo Cruz, Rio de Janeiro, Brazil.; 3 Cleveland Clinic Lerner College of Medicine, CWRU School of Medicine, Cleveland, Ohio, USA.; 4 Department of Orthopaedic Surgery and; 5 Department of Rheumatic and Immunologic Diseases, Cleveland Clinic, Cleveland, Ohio, USA.; 6 Department of Medicine, Kao Autoimmunity Institute, and Division of Rheumatology, Cedars-Sinai Medical Center, Los Angeles, California, USA.; 7 Department of Cancer Biology, Lerner Research Institute, Cleveland Clinic, Cleveland, Ohio, USA.; 8 Florida Research and Innovation Center, Cleveland Clinic, Port St. Lucie, Florida, USA.; 9 Respiratory Institute, Lerner Research Institute, Cleveland Clinic, Cleveland, Ohio, USA.; 10 Department of Pediatrics, Division of Pediatric Infectious Diseases, David Geffen School of Medicine at UCLA, Los Angeles, California, USA.

**Keywords:** Inflammation, Virology, Autoimmune diseases, Bone disease, Rheumatology

## Abstract

Patients with immune-mediated inflammatory diseases (IMIDs) like rheumatoid arthritis (RA) are at higher risk for severe COVID-19 and long-term complications in bone health. Emerging clinical evidence demonstrated that SARS-CoV-2 infection reduces bone turnover and promotes bone loss, but the mechanism underlying worsened bone health remains elusive. This study sought to identify specific immune mediators that exacerbated preexisting IMIDs after SARS-CoV-2 exposure. Plasma samples from 4 groups were analyzed: healthy, IMID only, COVID-19 only, and COVID-19 + IMID. Using high-throughput multiplexed proteomics, we profiled 1,500 protein biomarkers and identified 148 unique biomarkers in COVID-19 patients with IMIDs, including elevated inflammatory cytokines (e.g., IL-17F) and bone resorption markers. Long-term circulating SARS-CoV-2 ORF8, a virulence factor for COVID-19, was detected in the COVID + IMID group. RA was one of the most common IMIDs in our study. ORF8 treatment of RA-derived human osteoblasts (RA-hOBs) increased levels of inflammatory (*TNF*, *IL6*, *CCL2*) and bone resorption (*RANKL*/osteoprotegerin ratio) markers compared with healthy controls. Supernatants from ORF8-treated RA-hOBs drove the differentiation of macrophages into osteoclast-like cells. These findings suggest that SARS-CoV-2 exposure can exacerbate IMIDs through ORF8-driven inflammation and osteoclastogenesis, highlighting potential therapeutic targets for managing COVID-19–induced bone pathologies.

## Abstract 2

Patients with immune-mediated inflammatory diseases (IMIDs), like rheumatoid arthritis, face increased COVID-19 severity. SARS-CoV-2 ORF8 may exacerbate IMIDs, involving heightened inflammatory and bone-resorptive markers.

## Graphical abstract

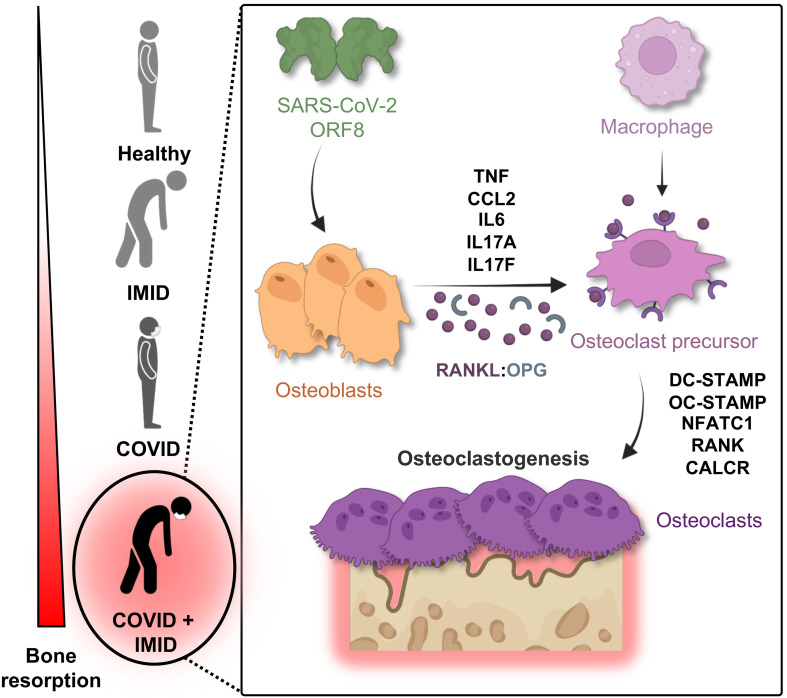

## Introduction

The massive outbreak of novel COVID-19 has led to its rapid global spread, resulting in a pandemic and an unprecedented global health crisis (1). Individuals with chronic immune-mediated inflammatory diseases (IMIDs), such as rheumatoid arthritis (RA), spondylarthritis, juvenile idiopathic arthritis (JIA), psoriatic arthritis (PsA), systemic lupus erythematosus (SLE), and psoriasis, have faced additional challenges during the COVID-19 pandemic. Given that SARS-CoV-2 infection is characterized by immune dysregulation, acute exacerbations among these individuals with IMID could negatively affect their quality of life and increase mortality (2). In fact, COVID-19 is a known risk factor for rheumatic disease flares, with IMID patients, particularly those with RA, demonstrating a 3-fold higher susceptibility to infection or death (3–8). While current literature has focused mainly on rheumatic flares after COVID-19 vaccination, inflammatory disease flares in patients with IMID following natural SARS-CoV-2 infection and their underlying mechanisms remain unexplored.

Several retrospective studies suggest that individuals with rheumatic diseases have an increased risk of SARS-CoV-2 infection, hospitalization, and COVID-19–related mortality compared with the general population (6, 9–12). While the relationship between SARS-CoV-2 infection and IMIDs is still being investigated, epidemiological studies have shown an association between SARS-CoV-2 infection and a wide range of autoimmune disorders, including inflammatory arthritis (13–15). Additionally, there are growing clinical reports of disease flares in specific rheumatic conditions, such as RA (3, 4), JIA (16), SLE (17, 18), psoriasis (19), and idiopathic inflammatory myopathies (20). Given the clinical relevance of these observations, we can draw mechanistic insights from viruses known to induce bone loss, such as chikungunya virus and dengue virus, as we advance our research on how SARS-CoV-2 disrupts bone health (21).

The SARS-CoV-2 genome encodes 29 viral proteins, including 4 structural proteins, 16 nonstructural proteins, and 9 accessory proteins (22). Among these viral proteins, the 121–amino acid ORF8 is unique as a secreted protein, featuring a signal sequence and an Ig-like domain (23). Notably, a naturally occurring SARS-CoV-2 variant with a 382-nucleotide deletion in the ORF8 transcription regulatory sequence, resulting in the loss of ORF8 expression, has been associated with milder COVID-19 symptoms (24, 25). Functional studies on ORF8 have revealed its immunomodulatory roles, including downregulation of MHC I and antagonism of IFN signaling (22). Moreover, recent findings suggest that ORF8 mimics IL-17 signaling in peripheral blood monocytes, potentially contributing to severe COVID-19 inflammation (26, 27).

While growing evidence shows that SARS-CoV-2 infection can exacerbate preexisting rheumatic conditions, the underlying mechanisms remain poorly understood. In this study, we aimed to investigate this phenomenon by analyzing 73 plasma specimens from patients using aptamer-based SomaScan technology to profile 1,500 protein biomarkers. Our findings revealed that COVID-19 patients with preexisting IMIDs exhibited elevated levels of inflammatory and osteoclastic bone factors. Notably, circulating SARS-CoV-2 ORF8 was higher in COVID-19 patients with IMIDs than those without IMIDs. We further demonstrated that SARS-CoV-2 ORF8 treatment enhanced the inflammatory and osteoclastogenic responses of primary human osteoblasts (hOBs) derived from RA patients (RA-hOBs) compared with those from healthy controls (H-hOBs). Moreover, supernatants from RA-hOBs markedly induced more osteoclast differentiation in murine bone marrow–derived macrophages (BMDMs) than those from H-hOBs. This study identifies a mechanism by which SARS-CoV-2 induces osteoclastogenesis, contributing to bone loss in post-COVID-19 sequelae.

## Results

### Demographic information and clinical characteristics of patients.

**Figure 1:**
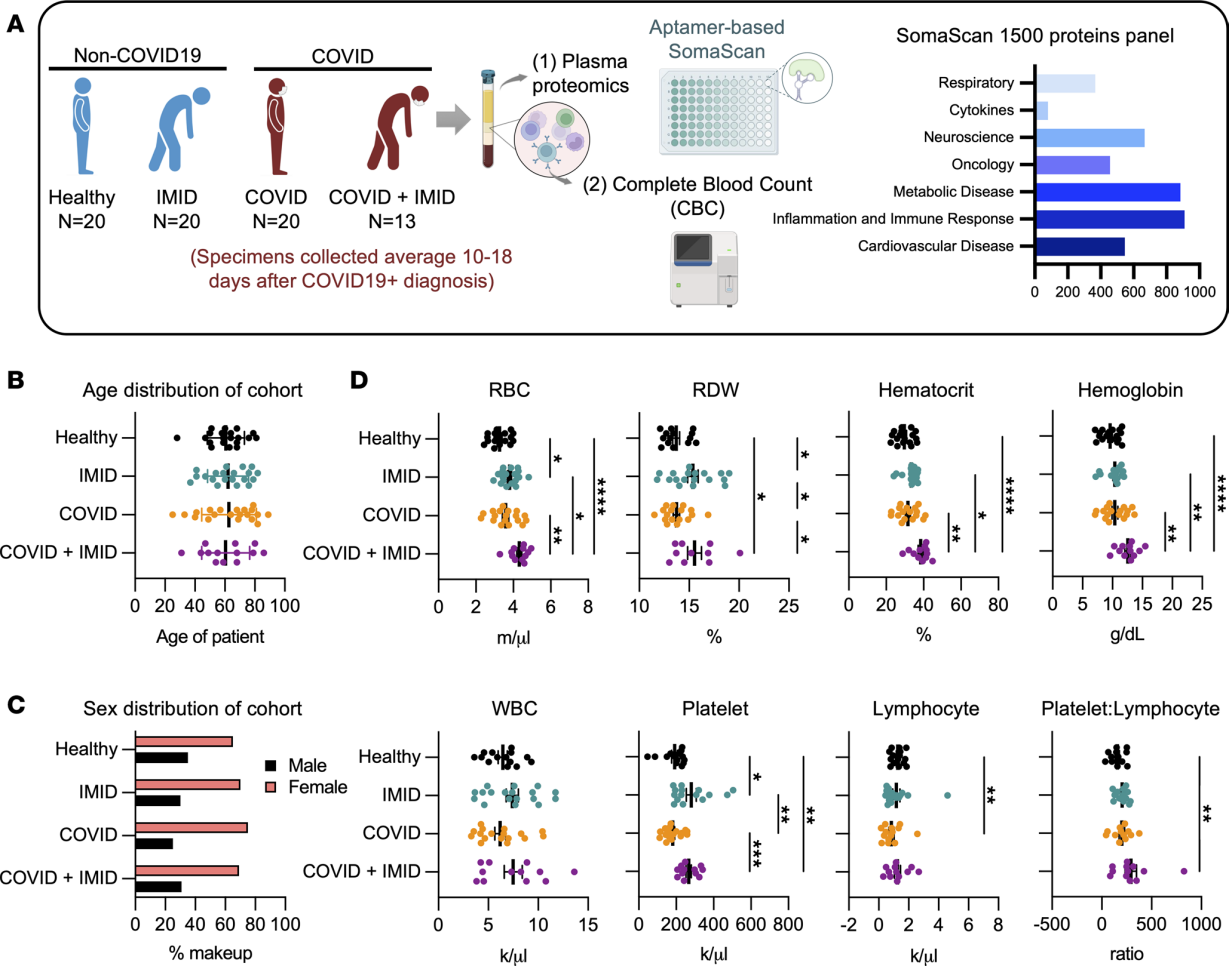
COVID-19 IMID clinical cohort study design and laboratory parameters. (**A**) Schematic of the clinical cohort and downstream workflow for blood specimens collected from (a) healthy (*n* = 20), (b) IMID (*n* = 20), (c) COVID (*n* = 20), and (d) COVID + IMID (*n* = 13). (**B**) Dot plot representing mean age distribution across the cohort. Data are represented as means ± SEM. (**C**) Bar plot of sex distribution across the cohort, representing the percentages of male participants (black bars) and female participants (red bars) in each group. (**D**) Dot plots showing CBC parameters analyzed from whole blood samples, including millions of RBC/μL, percentage of RDW, hematocrit percentage; hemoglobin levels (g/dL), thousands of WBC/μL, thousands of platelets/μL, thousands of lymphocytes/μL, and the ratio of platelet and lymphocyte counts. Data are represented as means ± SEM (*n* = 12–18 per group). Statistical analysis was performed using 1-way ANOVA, Kruskal-Wallis test, uncorrected Dunn’s posttest. Significant differences among groups are displayed with asterisks (*P < 0.05, **P < 0.01, ***P < 0.001, and ****P < 0.0001).

A total of 73 plasma specimens were retrieved from Cleveland Clinic Biorepository (CC-BioR), comprising 4 groups: (a) healthy controls with no history of COVID-19 or IMIDs (healthy, *n* = 20), (b) patients with confirmed IMIDs but no history of COVID-19 (IMID only, *n* = 20), (c) patients with confirmed COVID-19 but no history of IMIDs (COVID only, *n* = 20), and (d) patients with both IMIDs and COVID-19 (COVID + IMID, *n* = 13) (Table 1 and Figure 1A).

In the COVID group, blood samples were collected an average of 18 days after COVID-19 diagnosis (DAC), while in the COVID + IMID group, samples were collected an average of 10 DAC (Table 1). The 4 groups were matched for age and sex, with an average age of approximately 61 years, and with women making up 65%–71% of the participants in all groups (Table 1 and Figure 1, B and C). White participants were the majority (50%–60%), followed by Black participants (28%–40%). A minority of participants were multiracial, though none were in the IMID group (Table 1).

Regarding complete blood count (CBC) parameters, the IMID group had higher red blood cell (RBC) counts, red cell distribution width (RDW), and platelet levels compared with the healthy group (Table 1 and Figure 1D). The COVID group showed a reduced lymphocyte count relative to healthy controls (Table 1 and Figure 1D). Notably, the COVID + IMID group exhibited more pronounced alterations, including elevated RBC, RDW, hematocrit, hemoglobin, platelets, and platelet/lymphocyte ratio, compared with both healthy and COVID-only groups. Additionally, the COVID + IMID group had higher RBC, hematocrit, and hemoglobin when compared with the IMID group (Table 1 and Figure 1D).

Despite the varying types of IMIDs in the IMID and COVID + IMID groups, all conditions are characterized by dysregulated immune responses, leading to systemic inflammation and organ damage. The most common IMID conditions in both IMID and COVID + IMID groups were RA (14.81% and 16.67%, respectively) and SLE (14.81% and 16.67%, respectively) (Table 1). Other conditions in IMID and COVID + IMID groups included polymyalgia rheumatica (11.11% and 5.56%, respectively), scleroderma (7.41% and 11.11%), Raynaud’s disease (7.41% and 5.56%, respectively), Sjögren’s syndrome (7.41% and 16.67%, respectively), and granulomatosis with polyangiitis (3.70% and 5.56%, respectively). Additional conditions unique to specific groups are listed in Table 1.

### Elevated inflammatory and bone resorptive biomarkers in COVID-19 patients with preexisting IMIDs.

**Figure 2:**
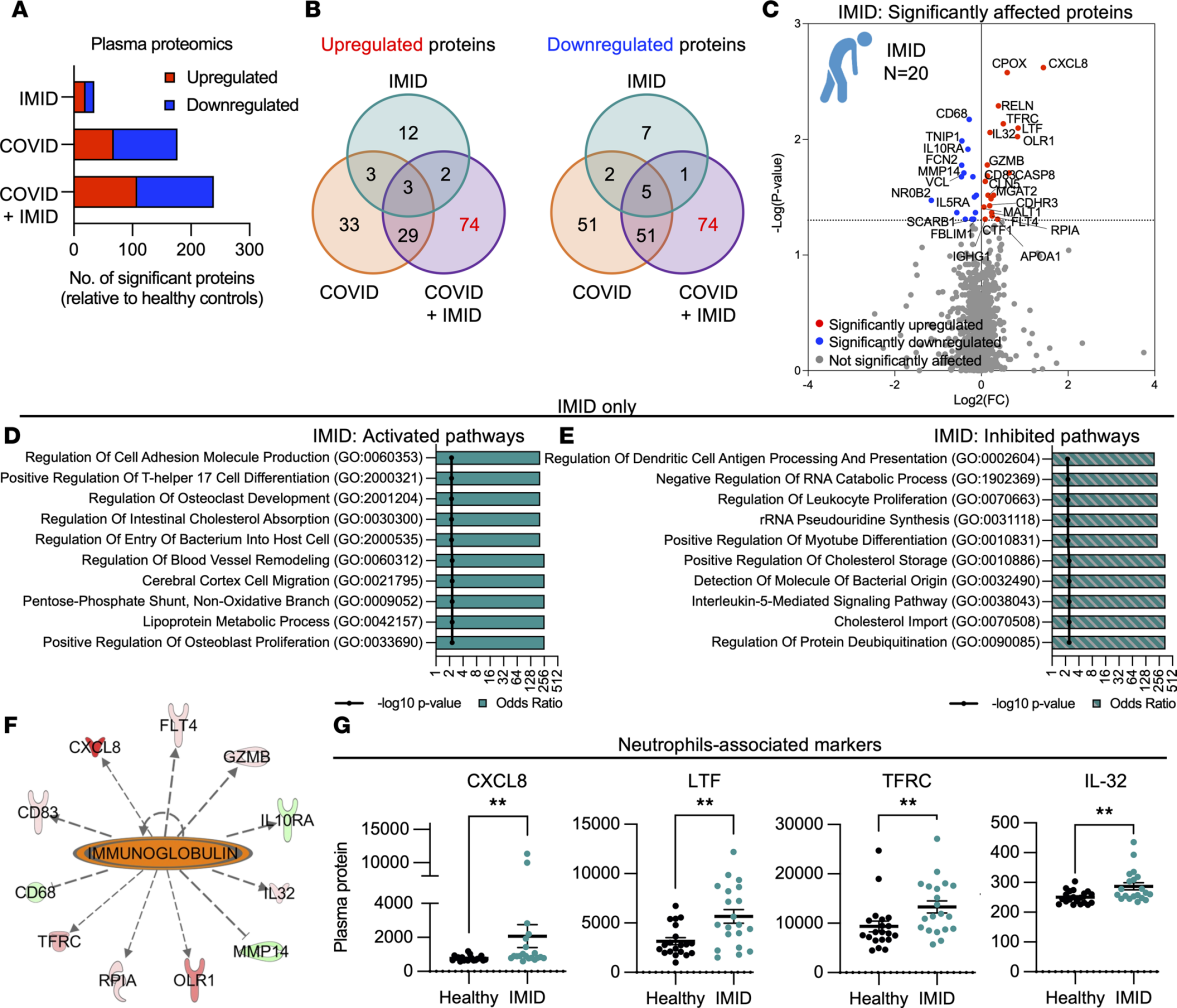
Patients with preexisting IMIDs exhibit minimal differences in basal immune status. (**A**) Significantly altered (*P* < 0.05) proteins in IMID, COVID, and COVID + IMID compared with healthy controls. (**B**) Quantitative comparison analysis of the number of downregulated and upregulated proteins in Venn diagrams of IMID, COVID, and COVID + IMID groups compared with healthy controls. (**C**) Volcano plot illustrating the proteins that are significantly altered (*P* < 0.05) in the IMID group. (**D**) Activated and (**E**) inhibited pathways in the IMID group. (**F**) Network analysis of the upstream regulator in the IMID group. (**G**) Dot plots showing plasma levels of pro-inflammatory proteins CXCL8, LTF, TFRC, and IL-32 in healthy and IMID groups. Data are represented as means ± SEM. *n* = 20. Statistical analysis was performed using Mann-Whitney *U* test. **P < 0.01.

To assess the impact of COVID-19 on patients with preexisting IMIDs, we used plasma samples from the retrospective patient cohort and performed SomaScan proteomics profiling across 1,500 proteins (Figure 1A). The high-throughput plasma proteomic analysis across all 4 patient groups revealed that the COVID + IMID group had the highest number of significantly differentially expressed proteins (DEPs) relative to healthy controls, with 108 upregulated and 131 downregulated proteins, compared with the IMID group (20 upregulated and 15 downregulated proteins) and the COVID group (68 upregulated and 109 downregulated proteins) (Figure 2, A and B).

Enrichment analysis of DEPs in the IMID group highlighted the activation of bone-associated pathways, such as osteoclast development and positive regulation of osteoblast proliferation. In contrast, pathways associated with the dendritic cell antigen processing and presentation, as well as leukocyte proliferation, were markedly inhibited in this group (Figure 2, C–E). Using Ingenuity Pathway Analysis (IPA), Ig was predicted as an upstream regulator in patients with IMID, suggesting an autoimmune bias in IMID conditions for the IMID group (Figure 2F). Notably, among the 20 upregulated proteins in the IMID group, we observed elevated expression of pro-inflammatory markers such as CXCL8, lactoferrin (LTF), transferrin receptor (TFRC), and IL-32, which are associated with neutrophil activation commonly observed in RA (28) (Figure 2G).

**Figure 3:**
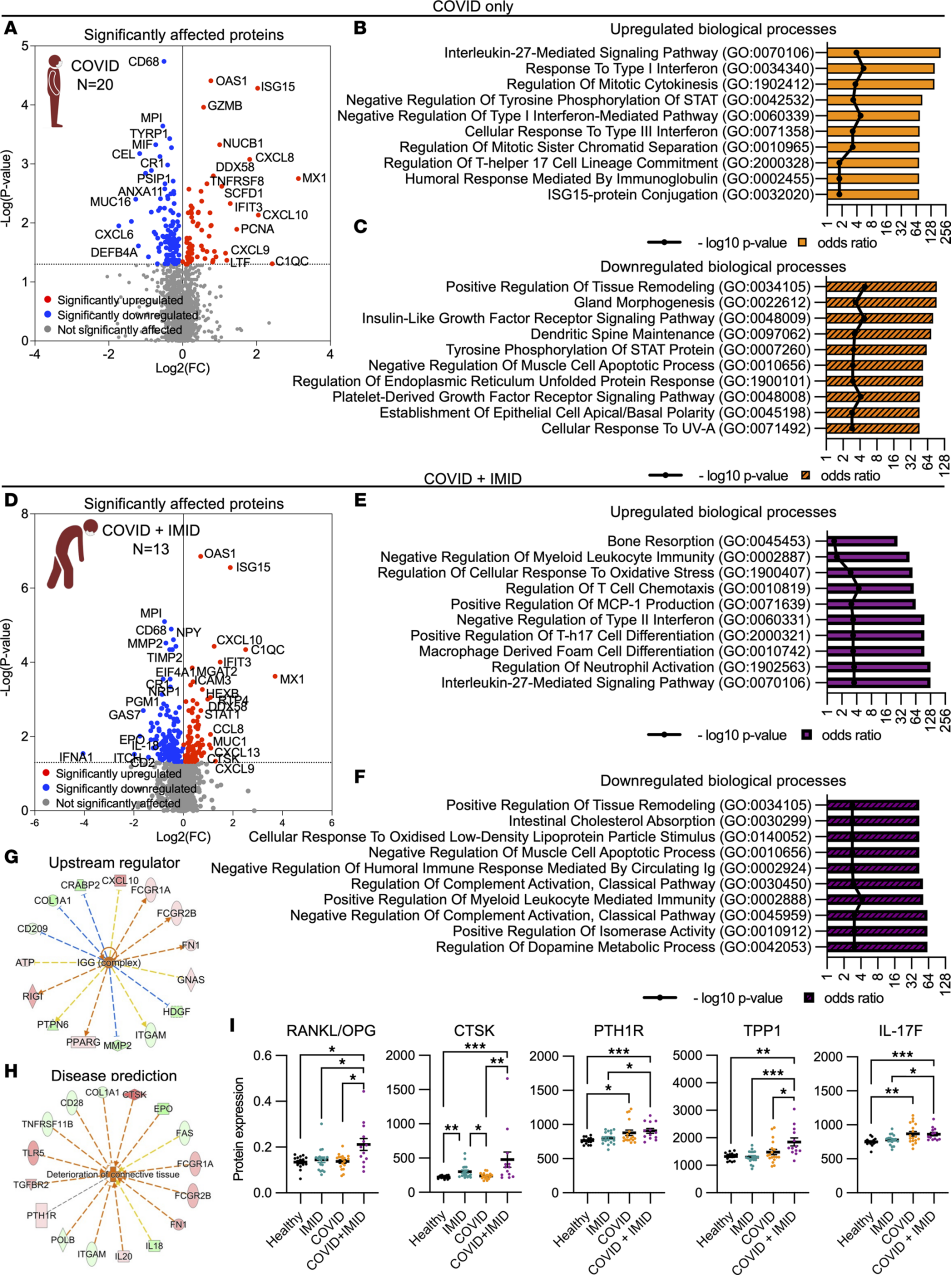
Immune dysregulation in COVID-19 patients with preexisting IMIDs. (**A**) Volcano plot illustrating the significantly altered proteins (*P* < 0.05) in the COVID group. (**B**) Upregulated and (**C**) downregulated biological processes in the COVID group. (**D**) Volcano plot illustrating the proteins that are significantly altered (*P* < 0.05) in the COVID + IMID group. (**E**) Upregulated and (**F**) downregulated biological pathways in COVID + IMID group. (**G** and **H**) Network analysis of upstream regulator (**G**) and disease prediction (**H**) in COVID + IMID group. (**I**) Dot plots of plasma levels of bone-remodeling cycle biomarkers, including ratio RANKL/OPG, CTSK, PTH1R, TPP1, and IL17F. Data are represented as means ± SEM (*n* = 12–18 per group). Statistical analysis was performed using 1-way ANOVA, Kruskal-Wallis test, and uncorrected Dunn’s posttest. Significant results are displayed with asterisks (*P < 0.05, **P < 0.01, and ***P < 0.001).

In contrast, the COVID group showed elevated levels of antiviral proteins, including 2′-5′-oligoadenylate synthetase 1 (OAS1), IFN-stimulated gene 15 (ISG15), IFN-induced protein with tetratricopeptide repeats 3 (IFIT3), DEAD-box helicase 58 (DDX58), and MX dynamin-like GTPase 1 (MX1), as well as pro-inflammatory proteins such as CXCL8, CXCL10, CXCL9, LTF, and complement C1q C chain (C1QC) (Figure 3A). Pathway analysis also predicted the activation of inflammatory cytokine IL-27 and antiviral signaling pathways, such as types I/III IFN and STAT signaling. Concurrently, there was a downregulation of pathways involved in cellular signaling, such as IGF receptor and PDGF receptor pathways (Figure 3, B and C).

Similarly, the COVID + IMID group exhibited pronounced expressions of inflammatory cytokines, such as CCL8, CXCL10, CXCL13, and CXCL9, along with bone factors, such as cathepsin K (CTSK) (Figure 3D). Indeed, further analysis of the upregulated proteins in the COVID + IMID group revealed the activation of bone resorption and CCL2 production, which is a crucial mediator in recruiting osteoclast progenitor cells (29) (Figure 3E). Additionally, several downregulated biological processes were associated with immune regulation, such as the negative regulation of the humoral immune response mediated by circulating Ig and the positive regulation of myeloid leukocyte-mediated immunity (Figure 3F). The COVID + IMID group exhibited a predicted upstream regulator effect of IgG, along with implications of connective tissue deterioration (Figure 3, G and H).

Osteoblasts regulate bone resorption through osteoprotegerin (OPG) production, which competes with RANK for RANKL, thereby inhibiting osteoclastogenesis. Consequently, the RANKL/OPG ratio is a clinical biomarker for bone loss. Indeed, we observed a significantly elevated RANKL/OPG ratio in the COVID + IMID group compared with the healthy, IMID, or COVID groups (Figure 3I). This increase was accompanied by increased levels of bone-remodeling proteins, including CTSK, parathyroid hormone 1 receptor (PTH1R), tripeptidyl peptidase 1 (TTP1), and IL-17F (Figure 3I). Most proteins showed no or weak correlation to DAC in the COVID + IMID group (Supplemental Figure 1A; supplemental material available online with this article; https://doi.org/10.1172/jci.insight.178820DS1), suggesting that DAC does not play a role in the immune dysregulation observed in patients from the COVID + IMID group. Collectively, these data indicate increased inflammation and bone resorption in COVID-19 patients with preexisting IMIDs.

### Plasma SARS-CoV-2 ORF8 correlates with enhanced inflammatory and dysregulated bone responses in patients with COVID-19 and IMIDs.

**Figure 4:**
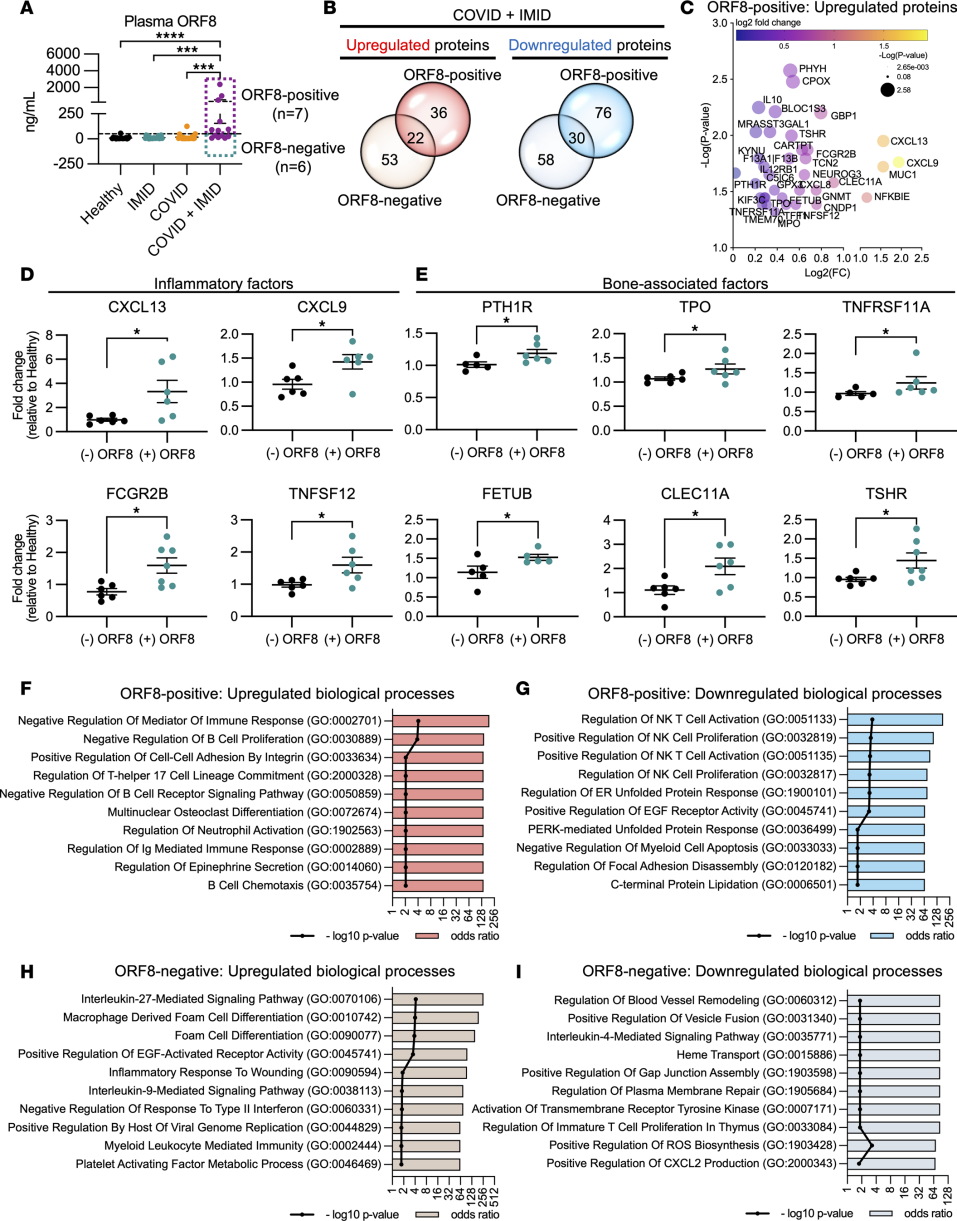
Plasma levels of SARS-CoV-2 ORF8 in COVID-19 patients with preexisting IMIDs correlate with enhanced inflammation and bone resorption. (**A**) Dot plots showing plasma levels of SARS-CoV-2 ORF8 detected in healthy, IMID, COVID, and COVID + IMID groups. Concentration > 50 ng/mL is considered ORF8 positive. Data are represented as means ± SEM (*n* = 13–20 per group). Statistical analysis was performed using 1-way ANOVA, Kruskal-Wallis test, and uncorrected Dunn’s posttest. ***P < 0.001 and ****P < 0.0001. (**B**) Venn diagram comparison of significantly altered (*P* < 0.05) plasma proteins among ORF8-negative (*n* = 6 per group) and ORF8-positive (*n* = 7 per group) COVID + IMID patients compared with healthy controls. (**C**) Bubble plot of significantly upregulated proteins in patients with ORF8-positive COVID + IMID. (**D** and **E**) Dot plots of fold-change (relative to healthy controls) of inflammatory factors CXCL13, CXCL9, FCGR2B, and TNFSF12 (**D**) and bone-associated factors PTH1R, TPO, TNFRSF11A, FETUB, CLEC11A, and TSHR (**E**) in patients with COVID + IMID belonging to ORF8-negative or ORF8-positive groups (*n* = 5–7 per group). (**F** and **G**) Biological processes upregulated (**F**) and downregulated (**G**) in patients with ORF8-positive COVID + IMID. (**H** and **I**) Biological processes upregulated (**H**) and downregulated (**I**) in patients with ORF8-negative COVID + IMID. Data are represented as means ± SEM. Statistical analysis was performed using Mann-Whitney *U* test. Significant results are displayed with asterisks (*P < 0.05).

Elevated serum levels of SARS-CoV-2 ORF8 have been reported in severe COVID-19 cases (27). In this study, we quantified the ORF8 levels in plasma from our cohort and examined its potential effects on the immunoproteomic profiles of patients with COVID-19. Plasma samples were considered ORF8 positive if ORF8 levels exceeded 50 ng/mL. Intriguingly, while only 5% (1 out of 20 patients) of the COVID group tested ORF8 positive, 54% (7 out of 13 patients) of the COVID + IMID group tested ORF8 positive, with plasma ORF8 ranging 80–2,400 ng/mL (Figure 4A). Notably, there was no correlation between ORF8 levels and DAC in the COVID + IMID group (Supplemental Figure 1B), indicating that the increased levels of circulating ORF8 were independent of the timing of COVID-19 diagnosis. Furthermore, we detected circulating ORF8 among COVID-19 patients with different IMIDs (Supplemental Figure 1C). Specifically, 3 out of the 7 ORF8-positive IMID patients expressed markedly higher circulating ORF8 (500–2,400 ng/mL), and these patients were associated with PsA, RA, Sjögren’s syndrome, giant cell arteritis, and pyoderma gangrenosum (Supplemental Table 1). Our findings suggest no association of specific IMID conditions to circulating ORF8 levels.

To further delineate immune profiles, we compared DEPs between ORF8-positive and ORF8-negative subgroups of COVID + IMID patients. We identified 36 upregulated and 76 downregulated DEPs exclusively in ORF8-positive samples (Figure 4B). In the ORF8-positive COVID + IMID group, upregulated inflammatory proteins included CXCL13, CXCL9, Fcγ receptor IIb (FCGR2B), and TNF superfamily member 12 (TNFSF12), as well as bone-associated factors PTH1R, thyroid peroxidase (TPO), TNFRSF11A, fetuin B (FETUB), C-type lectin domain containing 11A (CLEC11A), and thyroid-stimulating hormone receptor (TSHR) (Figure 4, C–E). Indeed, based on the upregulated proteins, the ORF8-positive COVID + IMID group has predicted activation of biological processes related to Th17 cell regulation, osteoclast differentiation, Ig-mediated immune responses, and B cell chemotaxis (Figure 4F). Conversely, downregulated processes primarily involved NK T cell activation (Figure 4G). In the ORF8-negative COVID + IMID patients, upregulated biological processes were related to IL-27 signaling and macrophage-derived foam cell differentiation, while downregulated processes were associated with blood vessel remodeling and heme transport (Figure 4, H and I). These findings suggest elevated plasma ORF8 levels in COVID-19 patients with IMIDs correlate with increased inflammation and bone dysregulation.

### SARS-CoV-2 ORF8 disrupts bone remodeling in human osteoblasts from patients with RA.

**Figure 5:**
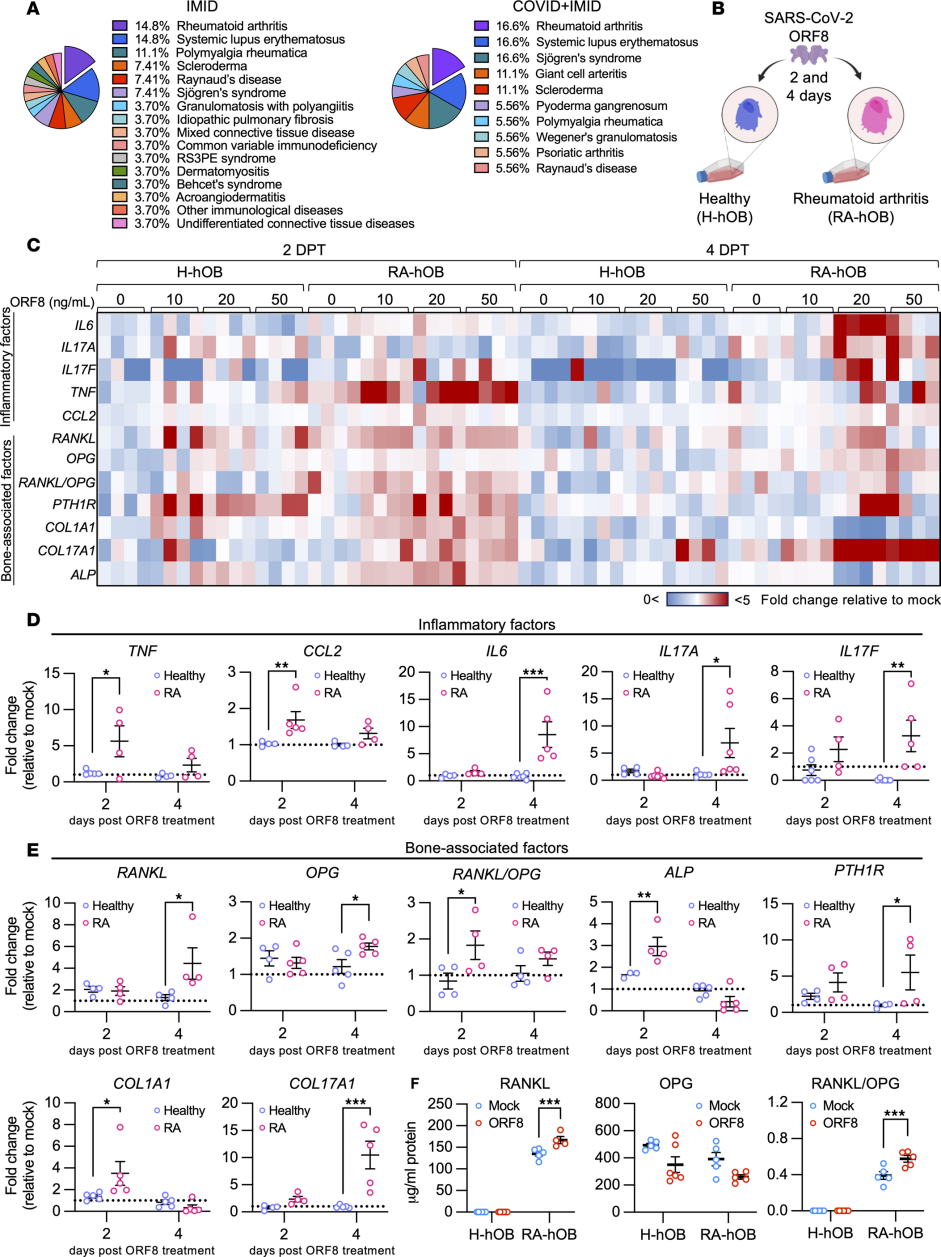
SARS-CoV-2 ORF8 treatment on primary human osteoblasts derived from patients with RA drives overt inflammation and dysregulates bone resorption markers. (**A**) Percentage of disease distribution in IMID and COVID + IMID groups. (**B**) Schematic representation of in vitro SARS-CoV-2 ORF8 stimulation in hOBs derived from healthy controls (H-hOBs) and patients with RA (RA-hOBs) upon SARS-CoV-2 ORF8 stimulation. Cells and supernatants were harvested at 2 days and 4 days posttreatment. (**C**) Heatmap representation of transcriptional profile of inflammatory factors *IL6*, *IL17A*, *IL17F*, *TNF*, and *CCL2* and bone-associated factors *RANKL*, *OPG*, *RANKL/OPG* ratio, *ALP*, *PTHR1*, *COL1A1*, and *COL17A1* in H-hOBs and RA-hOBs stimulated with 10 ng/mL, 20 ng/mL, or 50 ng/mL of purified SARS-CoV-2 ORF8, relative to mock-treated controls. (**D** and **E**) Transcriptional profile of inflammatory factors (**D**) and bone-associated factors (**E**) in H-hOBs and RA-hOBs stimulated with 20 ng/mL of purified SARS-CoV-2 ORF8, relative to mock-treated controls *n* = 4–6 per group. (**F**) Protein levels of RANK, OPG, and RANKL/OPG in the supernatant of mock controls and SARS-CoV-2 ORF8–stimulated H-hOBs and RA-hOBs. *n* = 4–6 per group. Data are represented as means ± SEM. Statistical analysis was performed using 2-way ANOVA and Bonferroni’s multiple comparisons test. Data were generated from 3 independent experiments. Significant differences among groups are displayed with asterisks (*P < 0.05, **P < 0.01, and ***P < 0.001).

Emerging clinical evidence suggests that SARS-CoV-2 infection may act as a potential trigger for flares in rheumatic diseases (3–5). To explore the association between COVID-19 and IMIDs, we categorized patients into 2 groups based on their diagnosis: IMID and COVID + IMID. In the IMID group, the most common diagnoses were RA, SLE, and polymyalgia rheumatica. A similar pattern emerged in the COVID + IMID group, where RA, SLE, and Sjögren’s syndrome were the most prevalent conditions (Figure 5A and Table 1). Given that bone resorption and bone homeostasis-disrupting pro-inflammatory factors were detected among patients with IMID (30), we investigated the role of ORF8 in modulating inflammation and bone homeostasis in IMIDs, particularly in RA, using primary H-hOBs and RA-hOBs.

We treated the primary H-hOBs and RA-hOBs with different concentrations of purified ORF8 (10, 20, and 50 ng/mL) or left them untreated (mock controls) for 2 and 4 days (Figure 5B). First, we compared gene expression levels in mock-treated cells to assess potential basal differences in inflammatory and bone markers between H-hOBs and RA-hOBs without ORF8 treatment. *CCL2*, *RANKL*, alkaline phosphatase (*ALP*), and collagen type I α1 chain (*COL1A1*) showed higher basal expression in RA-hOBs compared with H-hOBs (Supplemental Figure 2, A and B). Despite having higher basal levels of inflammatory and bone-associated genes, ORF8-treated RA-hOBs showed increased expression of inflammatory genes — *IL6*, *IL17A*, *IL17F*, *TNF*, and *CCL2* — as well as bone-associated genes — *RANKL*, *OPG*, *RANKL/OPG* ratio, *PTH1R*, *ALP*, *COL1A1*, and collagen type XVII α1 chain (*COL17A1*) — when compared with ORF8-treated H-hOBs (Figure 5C and Supplemental Figure 2, C and D). All ORF8 concentrations induced expression of inflammatory and bone resorption–related genes; however, we observed that 20 ng/mL of ORF8 for 2 days was the optimal condition to induce the majority of the inflammatory and bone-associated genes in RA-hOBs (Supplemental Figure 2, C and D).

Next, we compared the effects of ORF8 treatment (20 ng/mL) on H-hOBs and RA-hOBs. Interestingly, RA-hOBs exhibited significantly higher induction of pro-inflammatory genes (*TNF*, *CCL2*, *IL6*, *IL17A*, and *IL17F*) at 2 or 4 days compared with H-hOBs (Figure 5D). Additionally, transcriptional levels of bone-associated factors (*RANKL*, *OPG*, *RANKL/OPG* ratio, *ALP*, *PTH1R*, *COL1A1*, and *COL17A1*) were consistently upregulated in RA-hOBs but not in H-hOBs at either time point (Figure 5E). RANKL levels and the RANKL/OPG ratio were also higher in ORF8-treated RA-hOBs than in ORF8-treated H-hOBs, while the OPG protein level remained unchanged (Figure 5F).

**Figure 6:**
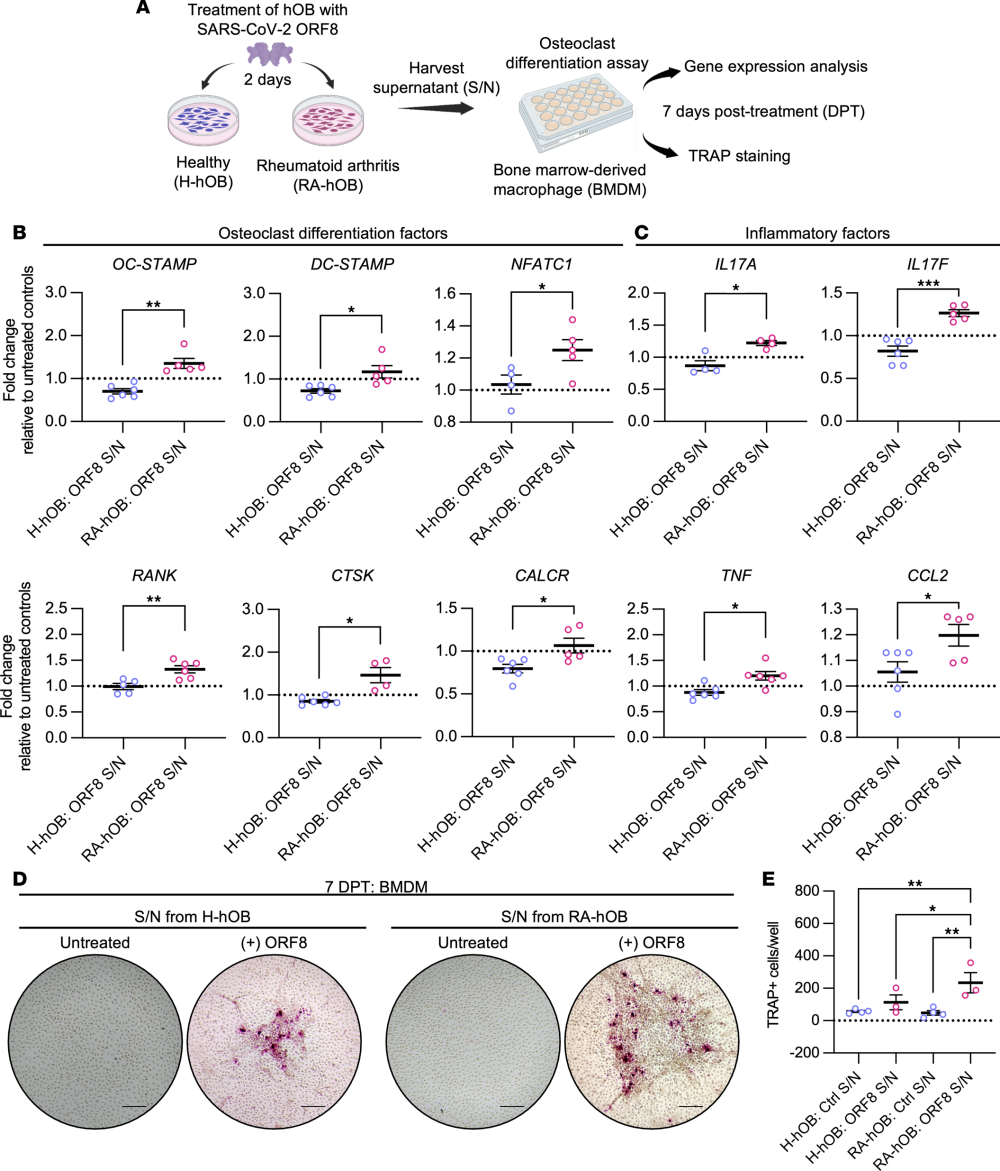
SARS-CoV-2 ORF8–treated osteoblasts drive osteoclastogenesis in BMDMs. (**A**) Murine BMDMs were treated for 7 days with supernatants from H-hOBs or RA-hOBs stimulated with or without purified SARS-CoV-2 ORF8 (20 ng) for 2 days. (**B**) Transcriptional profile of osteoclast differentiation markers *OC-STAMP*, *DC-STAMP*, *NFATC1*, *RANK*, *CTSK*, and *CALCR* and (**C**) inflammatory factors *IL17A*, *IL17F*, *TNF*, and *CCL2* in murine BMDMs. *n* = 4–6 per group. (**D**) Representative images of TRAP staining in murine BMDMs from 3 independent experiments. Scale bar = 150 μm. (**E**) Quantification of the TRAP+ cells per well. *n* = 3–4 per group. Data were analyzed using (**B** and **C**) Welch’s *t* test or (**D**) ordinary 1-way ANOVA and Fisher’s least significant difference test. Data are represented as means ± SEM. Significant differences among groups are displayed with asterisks (*P < 0.05, and **P < 0.01).

Fundamentally, we investigated the pro-osteoclastogenic effects of ORF8-treated hOBs by treating murine BMDMs, which serve as osteoclast precursor cells, with supernatants derived from ORF8-stimulated hOBs (Figure 6A). Interestingly, BMDMs treated with supernatant from ORF8-stimulated RA-hOBs exhibited enhanced expression of key osteoclast differentiation markers— osteoclast stimulatory transmembrane protein (*OC-STAMP*), dendrocyte expressed 7 transmembrane protein (*DC-STAMP*), nuclear factor of activated T cells 1 (*NFATC1*), *RANK*, *CTSK*, and calcitonin receptor (*CALCR*) — compared with BMDMs treated with supernatants from ORF8-stimulated H-hOBs (Figure 6B). Similarly, inflammatory factors, such as *IL17A*, *IL17F*, *TNF*, and *CCL2*, were significantly upregulated compared with the control group (Figure 6C). Furthermore, tartrate-resistant acid phosphatase (TRAP) staining revealed increased TRAP-positive osteoclast-like cell formation following exposure to supernatant from ORF8-treated RA-hOBs (Figure 6, D and E). Overall, these findings suggest that SARS-CoV-2 ORF8 acts as a pro-osteoclastogenic factor, promoting inflammation and osteoclastogenesis in COVID-19 patients with preexisting RA.

## Discussion

In May 2023, WHO declared the conclusion of the 4-year COVID-19 pandemic as a public health emergency of international concern (31). Throughout the pandemic, numerous studies have consistently reported a reduction in bone mineral density among hospitalized patients with severe COVID-19 (32, 33). Moreover, over 24% of long COVID-19 patients have reported bone pain up to 7 months after the onset of COVID-19 (34), suggesting that SARS-CoV-2 infection can disrupt bone health. Over the past 38 months, nearly 800 million confirmed cases of COVID-19 have been reported globally (35). Reports of patients with preexisting IMIDs, such as RA, experiencing worsening symptoms after SARS-CoV-2 infection have emerged (3–5), yet the underlying mechanisms triggering these exacerbations remain unclear.

In this study, we found that COVID-19 patients with preexisting IMIDs demonstrated marked hematological alterations compared with the control cohorts, indicating increased risks of immunological exacerbations among these individuals after COVID-19 infection. Patients with COVID-19 exhibit a range of RBC abnormalities, including impaired erythropoiesis and altered RBC morphology (36). We also consistently observed increased RBC counts, hemoglobin, and hematocrit levels in COVID + IMID patients. Compared with the healthy and COVID groups, the elevated RDW in both IMID and COVID + IMID groups also suggests persistent inflammation and potential complications in the COVID + IMID cohort. Our findings extend the findings of prior work, which demonstrated increased RDW during inflammation among patients with RA (37) and an elevated risk of adverse cardiac events in patients with psoriasis and PsA (38). These alterations are likely compensatory responses following an initial decrease in RBC counts during the acute phase of SARS-CoV-2 infection (36). Further, population studies showed that COVID-19 patients with preexisting IMIDs are at higher risk of hospitalization or death compared with the general population (39). Although the severity of COVID-19 in our patient groups remains undetermined, the observed increase in the platelet/lymphocyte ratio in COVID + IMID patients, a marker associated with COVID-19 severity and mortality (40), suggests that these patients may have experienced more severe COVID-19 complications.

IMIDs are a diverse group of chronic diseases characterized by dysregulated immune responses, reduced quality of life, and increased mortality risk. In this cohort, Ig was identified as an upstream regulator of the altered immunoproteomics profiles in both IMID and COVID + IMID groups, suggesting an autoimmune-biased immune response. Indeed, the majority of the patients in our study were diagnosed with RA, SLE, or polymyalgia rheumatica. Interestingly, marked upregulation of bone resorption pathways was observed in the COVID + IMID group, accompanied by elevated markers such as RANKL/OPG, CTSK, PTH1R, and TPP1, indicative of enhanced osteoclastogenesis. Previous studies have reported persistent bone pain in patients with COVID-19 months after the acute phase (34), while animal models have demonstrated severe bone loss driven by SARS-CoV-2–induced osteoclast formation (41–43). Importantly, although SARS-CoV-2 RNA was undetected in bone tissue, a substantial increase in osteoclast numbers was observed even after the resolution of active viral replication (2 weeks after infection). This suggests that the virus may induce bone loss through inflammatory mechanisms rather than direct infection of bone cells (42). A marked increase in circulating RANKL was recently observed in COVID-19 patients with periodontal diseases for up to 100 days following COVID-19 diagnosis (44), consistent with our findings among the COVID + IMID cohort. These data raise concerns about the potential activation of bone resorption and exacerbation of systemic inflammation even after COVID-19 infection. With the identification of SARS-CoV-2 ORF8 among COVID-19–infected individuals with preexisting IMIDs, COVID-19 likely amplifies postinfection inflammatory responses, leading to more severe symptomatic outcomes. Thus, our findings highlight SARS-CoV-2 ORF8 as a rational biomarker and critical viral factor contributing to this heightened pro-inflammatory osteoclastogenic response observed in patients with IMID patients.

The IL-17 cytokine family, especially IL-17A to IL-17F, is well established in promoting osteoclastogenesis by upregulating RANKL expression and skewing the RANKL/OPG ratio (45, 46). In RA models, IL-17A is implicated in disease pathogenesis, but clinical trials targeting IL-17A alone yielded modest effects, suggesting that other IL-17 family members, such as IL-17F, may also be involved (47). Here, we observed elevated IL-17F levels in both the COVID only and COVID + IMID groups compared with healthy and IMID only patients, indicating the active involvement of IL-17F in the inflammatory response to SARS-CoV-2 infection. We also found abundant circulating SARS-CoV-2 ORF8 among half of the COVID + IMID cohort but rarely in the COVID only group. Given its mimicry of the IL-17 in hyperinflammatory cytokine release (26, 27), we hypothesized that ORF8 is critical in increased inflammation and osteoclastogenesis. When RA-hOBs were stimulated with ORF8, the deleterious impact of the SARS-CoV-2 infection, as reflected by the upregulation of pro-inflammatory and bone remodeling–associated genes, corroborates with prior findings (48–52) and provides a direct link between COVID-19 and bone loss (53, 54). We further validated these findings when supernatant from ORF8-treated RA-hOBs significantly induced osteoclast formation in BMDMs (Figure 6, D and E), strongly implicating ORF8 as a critical bone remodeling factor in RA. Overall, the combined exacerbated inflammatory responses, Th17 cell expansion, and enhanced osteoclastogenic activity among the COVID + IMID cohort collectively suggest an ORF8-mediated inflammation and heightened risk of systemic bone loss among SARS-CoV-2–infected individuals with preexisting IMIDs. Our current findings underscore the need to investigate the mechanism of ORF8 in inducing osteoclastogenesis to potentially design therapeutic strategies to mitigate severe outcomes in skeletal health.

Given that the current work is not within the pretext of a clinical cohort study, the specimens were obtained retrospectively from the biorepository and lacked detailed patient medical histories, such as COVID-19 disease severity, medication use, and COVID-19 vaccination status. In addition, our cohort included a diverse array of IMID conditions for which we do not have information on whether patients were experiencing autoimmune flares at the time of sample collection. Nonetheless, we demonstrated that patients with heterogenous IMIDs characterized by immune response dysregulation and baseline inflammation exhibited COVID-19 as a catalyst for enhanced inflammatory processes and disease outcomes. Future cohort studies focusing on specific rheumatic IMIDs, such as RA or PsA, will be crucial to better assess the impact of COVID-19 on IMID disease flares.

In summary, our data highlighted that SARS-CoV-2 infection amplifies inflammation and exacerbates osteoclastogenesis in patients with IMIDs. The viral ORF8 protein, acting as an IL-17 mimic, may drive bone resorption via IL-17–associated mechanisms. These findings shed light on the long-term post-COVID-19 sequelae in patients with preexisting inflammatory conditions, including bone loss. Targeting circulating ORF8 presents a promising therapeutic strategy for mitigating COVID-19–mediated arthritic exacerbations and bone damage in patients with IMIDs. These findings enhance our understanding of the interplay between SARS-CoV-2 infection and IMIDs, offering potential avenues for targeted therapeutic interventions aimed to improve patient outcomes.

## Methods

### Sex as a biological variable.

Both male and female participants were included across all 4 groups: (a) healthy controls, (b) IMID only, (c) COVID only, and (d) COVID + IMID. Women comprised 65%–71% of each group, reflecting the typical higher prevalence of IMIDs in women. The study was not designed to assess sex-specific differences, but sex was considered to ensure generalizability.

### Study cohort.

In this study, we utilized a retrospective clinical cohort of adult patients whose specimens were previously deposited in the CC-BioR during the COVID-19 pandemic between March 2020 and May 2021. The patient cohort included the following groups: (a) healthy controls, (b) IMID only, (c) COVID only, and (d) COVID + IMID. All 4 patient groups were age and sex matched. SARS-CoV-2–positive patients in the COVID and COVID + IMID groups were diagnosed with COVID-19 by nasopharyngeal SARS-CoV-2 quantitative real-time PCR (qRT-PCR). As inclusion criteria, blood specimens were collected an average of 18 DAC in the COVID only group and 10 DAC for the COVID + IMID group. Patients from IMID only and COVID + IMID groups were assigned with systemic connective tissue disorders International Classification of Diseases codes (M30–M36). CBC was conducted on all blood specimens collected. Plasma samples were isolated from whole blood specimens and stored at –80°C.

### Plasma proteomics profiling.

Plasma samples were analyzed using the SomaScan assay (SomaLogic). Briefly, the SomaScan assay contains 1,500 aptamers that recognize specific protein antigens. After binding, the levels of protein-aptamer complexes are quantified using next-generation sequencing (55). Raw data in.adat format were read into the R environment with the readat R package (56). Fold-change was calculated by first averaging the expression of each group and then using the following equation: (IMID only or COVID only or COVID + IMID expression)/(healthy expression). This formula accounts for negative expression values generated during the initial log_2_ conversion and normalization. Significant DEPs were determined by Welch *t* tests or Wilcoxon rank-sum tests using the R base package, *t* test, considering fold-change ≥ 2 and FDR-adjusted *P* value < 0.05.

### SARS-CoV-2 ORF8 ELISA.

Plasma samples were analyzed with the SARS-CoV-2 ORF8 ELISA as described previously (27). Briefly, Microlon 96-well polystyrene plate (Greiner Bio-One) was coated with 1 μg/mL of mouse monoclonal anti–SARS-CoV-2 ORF8 (R&D Systems, Bio-Techne, catalog Mab10820, clone 1041422) overnight at 2°C–8°C. Unbound antibody was washed 2 times with 300 μL/well of Tris-buffered saline 0.05% Tween-20 (TBST, pH 8.0). The plate was then blocked with 300 μL/well of 3% BSA TBST buffer for 1 hour at room temperature. The standard curve consisted of the SARS-CoV-2 ORF8 with 10-fold serial dilution with 3% BSA TBST buffer (10,000 ng/mL to 0.001 ng/mL). After washing the plates twice, 100 μL/well of the standard curve, biospecimens, or only 3% BSA TBST buffer (blank) were applied in duplicates and incubated for 2 hours at room temperature. Plates were washed 4 times and incubated for 1 hour at room temperature with 1:500 of polyclonal anti-ORF8 antibody (GeneTex, catalog GTX135591) previously biotinylated using Biotinylation kit/Biotin conjugation kit (Fast, type A)-Lightning-Link (Abcam, catalog ab201795). Finally, plates were washed 6 times and incubated with TMB Substrate Reagent Set (OptEIA, BD Biosciences). Within 10–30 minutes, the reactions were stopped with 100 μL/well of 2N sulfuric acid and read at 450 nm using the plate reader Varioskan Lux. Data were acquired with the Skanlt microplate reader software (Thermo Fisher Scientific). The levels of SARS-CoV-2 ORF8 in ng/mL for each sample were calculated by interpolation with the standard curve using 4 parameters of logistic regression, with a reduction of blank wells at the GraphPad Prism 9.0 software. The ELISA limit of detection was set at 40 ng/mL ORF8.

### Primary cell cultures.

Primary hOBs were isolated from the bone of healthy adults (Cell Applications, Inc., catalog 406-05a) or patients with RA (Cell Applications, Inc., catalog 406RA-05a). Primary hOB cells were cultured in Osteoblast Growth Medium (Cell Applications, Inc., catalog 416-500) at 37°C and 5% CO_2_. All media were further supplemented with 10% fetal bovine serum (FBS) (Biowest USA) and 1% penicillin-streptomycin (P-S).

### ORF8 stimulation of H-hOB and RA-hOB.

Recombinant ORF8 pIRES-puro plasmid was generated in-house as described (27), then transiently transfected into HEK293T cells (American Type Culture Collection), and the ORF8-containing supernatants were concentrated and purified as previously described (27). Primary H-hOBs and RA-hOBs were seeded in 24-well plates at a density of 1 × 10^5^ cells per well. At 24 hours later, cells were treated with 10 ng, 20 ng, or 50 ng of purified ORF8 in a final volume of 1 mL of osteoblast growth medium supplemented with 10% FBS and 1% P-S. The same cell culture medium was added in mock controls. Cells were cultured at 37°C with 5% CO_2_. At 24 and 48 hours after ORF8 stimulation, cell supernatants were collected for ELISA, and cells were washed with PBS and collected in TRIzol (Invitrogen, catalog 15596018) for RNA extraction.

### RNA extraction and qRT-PCR.

Total RNA extractions from hOBs and BMDMs were performed using TRIzol Reagent and RNeasy Mini Kit (QIAGEN, catalog 74106), respectively, following the manufacturer’s instructions. The RNA concentration was determined using a spectrophotometer (NanoDrop 1000, Thermo Fisher Scientific). Extracted total RNA was transcribed using iScript cDNA synthesis kit for hOBs (Bio-Rad, catalog 1708891BUN) or iScript Select cDNA Synthesis Kit for BMDMs (Bio-Rad, catalog 1708897BUN), according to the manufacturer’s instructions. Quantitative PCR was performed using SsoAdvanced Universal SYBR Green Supermix (Bio-Rad, catalog 1725272) following manufacturer protocol. Gene expression levels for each sample were normalized to GAPDH, and fold-changes relative to mock or untreated samples were calculated with the ΔΔCt method. The fold-change for each gene was calculated as 2^-ΔΔCt^. Primer sequences used for qRT-PCR can be found in Supplemental Table 2.

### RANKL and OPG ELISA.

At 48 hours after ORF8 stimulation, supernatants from H-hOBs and RA-hOBs were collected, and the concentrations of RANKL (catalog DY626) and OPG (catalog DY805) were determined using human ELISA development kits from R&D Systems, Bio-Techne. The assays were performed according to the manufacturer’s instructions.

### BMDM isolation and TRAP staining.

Primary BMDMs and osteoclast cultures were prepared as previously described with slight modifications (57). BMDMs were extracted from the femora and tibiae of C57BL/6 mice (The Jackson Laboratory) and cultured in α–minimum essential medium containing 10% FBS, 1% P-S, 1% Glutamax Supplement (Gibco, catalog 35050061), and 25 ng/mL of recombinant human macrophage colony-stimulating factor (R&D Systems, Bio-Techne, catalog 216-MCC) for 6 days. The cells were subsequently treated with supernatants collected from H-hOBs and RA-hOBs 48 hours after ORF8 treatment for 7 days. For TRAP staining, cells were fixed and stained using a TRAP staining kit (Cosmo Bio LTD, catalog PMC-AK04F-COS) according to the manufacturer’s instructions. The images were captured using ECHO (BICO) Revolve microscope.

### Statistics.

Comparisons between 2 groups were determined by Mann-Whitney *U* tests, and when using more than 2 groups, the comparisons were conducted using 1-way ANOVA, Kruskal-Wallis with Dunn’s posttests, and 2-way ANOVA with Bonferroni’s multiple comparisons test using GraphPad Prism v9.0, considering a *P* value less than 0.05 significant. Enrichment analysis of the canonical pathways of the significant DEPs was conducted using IPA (QIAGEN), National Center for Advancing Translational Sciences BioPlanet, or STRING database.

### Study approval.

The cohort study and human sample collection were approved by the Cleveland Clinic Institutional Review Board (IRB-221019) and Institutional Biosafety Committee. Written informed consent for study participation was obtained for all participants before enrollment.

### Data availability.

Values for all data points in graphs are reported in the Supporting Data Values file. This paper does not report original code. Any additional information required to reanalyze the data reported in this paper is available upon request.

## Author contributions

SSF and WC conceived the study and designed the experiments. IM and TA performed all key experiments and bioinformatic data analyses. IM, TA, SSF, and WC prepared the figures and drafted the original and revised manuscript. CCSC, NMM, CO, DFM, GMS, and BLS contributed to experiments, data interpretation, and analyses. AV, CMC, ZV, and SAAC assisted with experiments and provided tools and resources. YC, YJC, SC, XW, LHC, MEH, JUJ, and NSP provided expertise, interpreted results, and commented on the manuscript. IM, TA, CCSC, NMM, CO, AV, DFM, GMS, BLS, CMC, YJC, SC, YC, XW, ZV, SAAC, KNS, LHC, MEH, JUJ, NSP, SSF, and WC contributed to editing the manuscript and approved the final version for submission. Authorship order among co–first authors was determined based on IM’s significant role in drafting the initial manuscript and coordinating key aspects of data analysis.

## Supplementary Material

Supplemental data

Supporting data values

## Figures and Tables

**Table 1:**
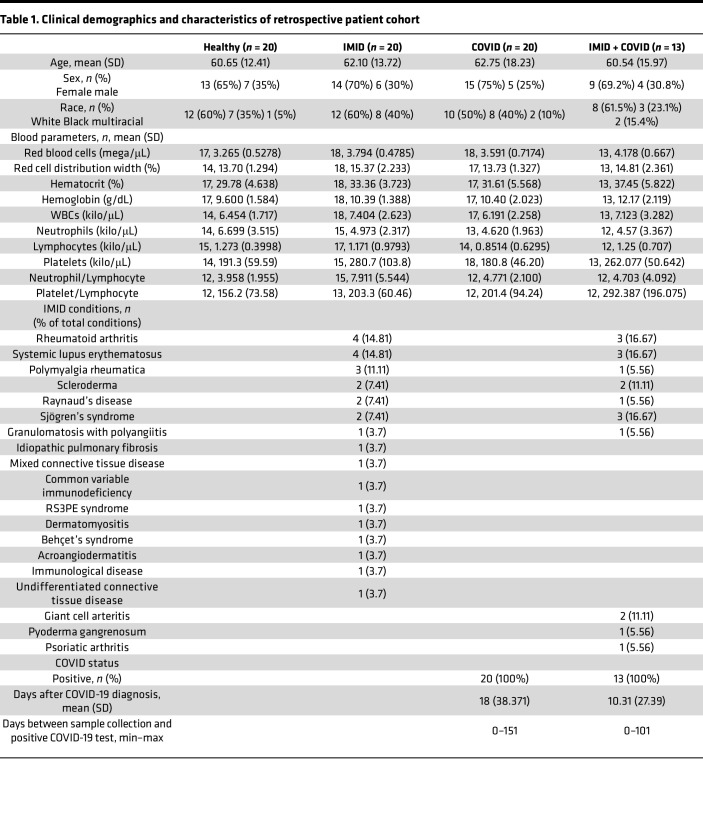
Clinical demographics and characteristics of retrospective patient cohort

## Version 1 12/20/2024

Electronic publication
