# Supplementary material for: SARS-CoV-2 ORF8 drives osteoclastogenesis in preexisting immune-mediated inflammatory diseases
Source: JCI Insight. 2024 Dec 20;9(24):e178820. doi: 10.1172/jci.insight.178820 (PMC11665583; doi:10.1172/jci.insight.178820)
Supplement: Supplemental data [file jciinsight-9-178820-s031.pdf]

Supplemental Figures and Tables

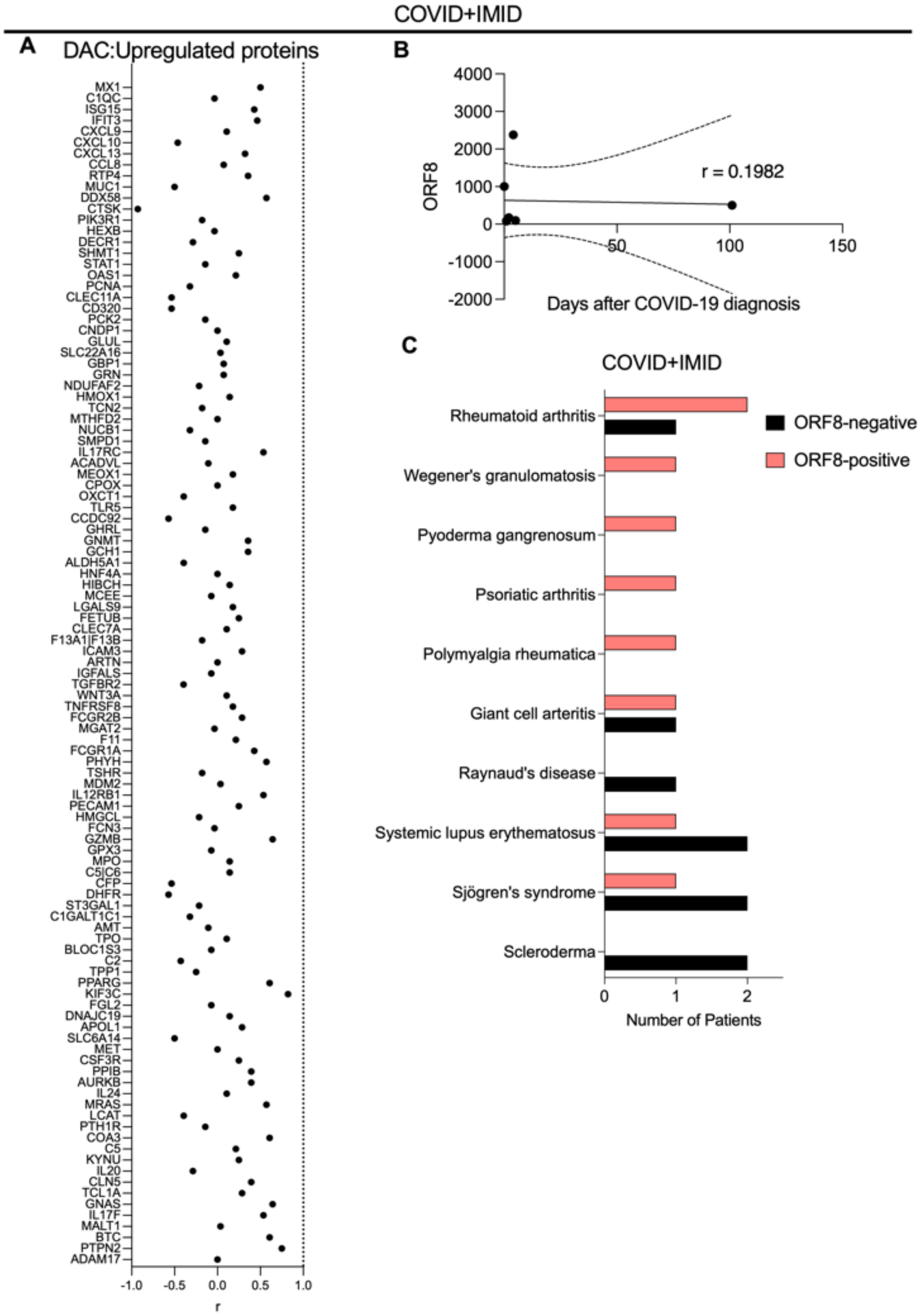

Supplemental figure 1. Correlation between protein levels, time of collection, ORF8 levels and IMID conditions. (**A** and **B**) Spearman's correlation analysis between days after COVID-19 diagnosis (DAC) with significantly upregulated proteins (**A**) and ORF8 (**B**) in the COVID+IMID group. (**C**) IMID conditions and ORF8 positivity in COVID+IMID group.

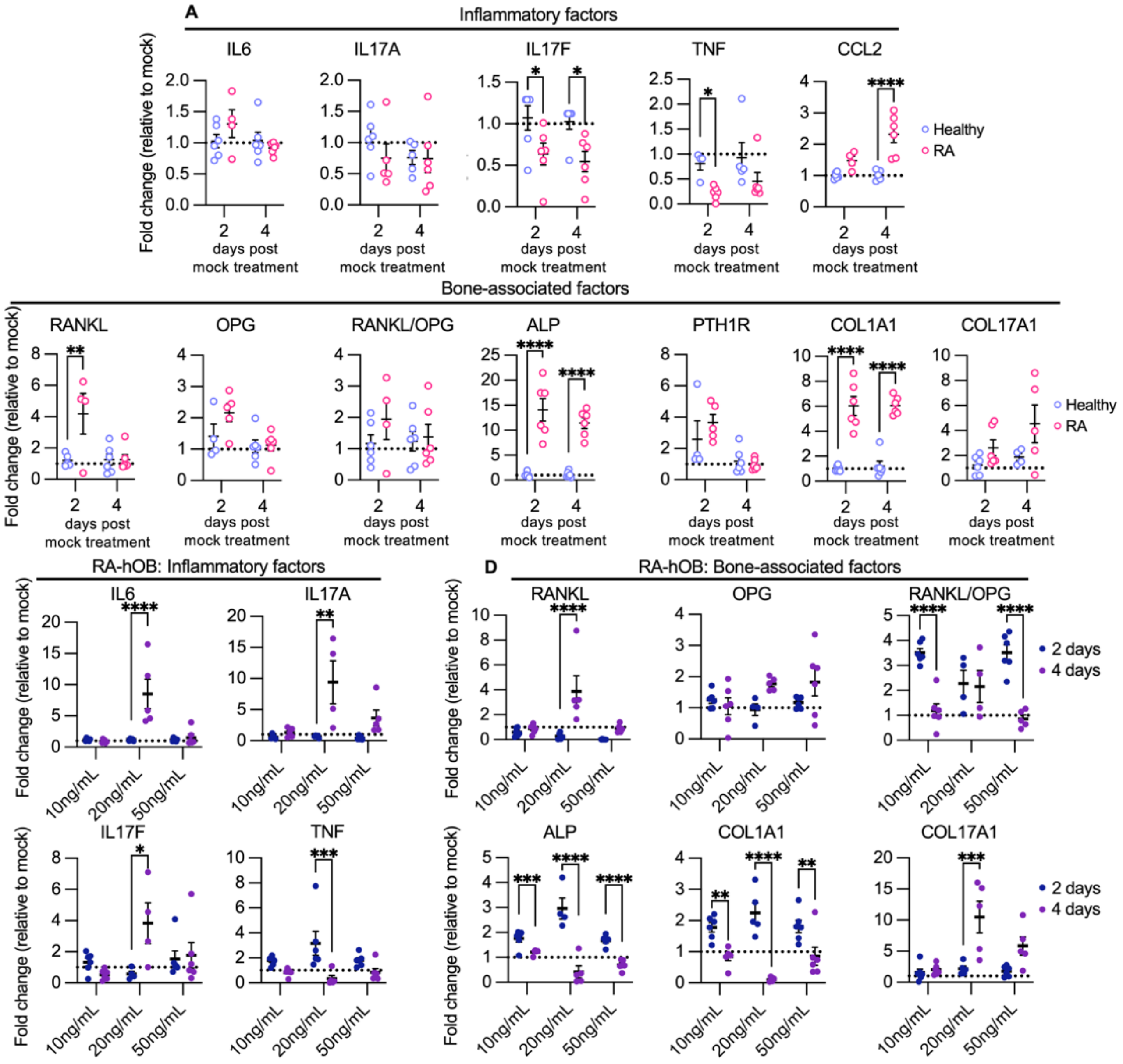

Supplemental figure 2. SARS-CoV-2 ORF8 dose-dependent effect in RA osteoblasts. (**A and B**) Transcriptional profile of inflammatory factors (**A**) and bone-associated factors (**B**) in H-hOBs and RA-hOBs treated with media (mock-treated controls) n = 4–6 per group. (**C and D**) Transcriptional profile of inflammatory factors (**C**) and bone-associated factors (**D**) in H-hOBs and RA-hOBs stimulated with 20 ng/mL of purified SARS-CoV-2 ORF8, relative to mock-treated controls n = 4–6 per group. Data are represented as means ± SEM. Statistical analysis was performed using two-way ANOVA, and Bonferroni's multiple comparisons test. Data were generated from three independent experiments. Significant differences among groups are displayed with asterisks (*P<0.05, **P<0.01, ***P<0.001 and ****P<0.0001).

Supplemental Table 1. Summary of types of IMID conditions and ORF8 positivity from COVID-19 with IMID cohort

IMID Conditions	COVID + IMID Patients												
	#1	#2	#3	#4	#5	#6	#7	#8	#9	#10	#11	#12	#13
Giant cell arteritis		✓						✓					
Polymyalgia rheumatica							✓						
Psoriatic arthritis	✓												
Pyoderma gangrenosum			✓										
Raynaud’s disease											✓		
Rheumatoid arthritis	✓					✓							✓
Scleroderma									✓		✓		
Sjögren's syndrome	✓									✓		✓	
Systemic lupus erythematosus				✓								✓	✓
Wegener's granulomatosis					✓								
ORF8 positivity	+	+	+	+	+	+	+	-	-	-	-	-	-
ORF8 level (ng/mL) [A]	2376.33	1000.66	504.79	173.21	95.40	90.15	84.85	33.11	31.13	23.17	14.83	8.10	7.24

[A] Samples with >50 ng/mL ORF8 detected were considered ORF8 positive.

Supplemental Table 2. qPCR list of primers used in this study		
**Gene**	**Sequence (5' - 3')**	**Species**
GAPDH-F	ACCAGGTGGTCTCCTCTGAC	human
GAPDH-R	TGTAGCCAAATTCGTTGTCATACC	human
IL6-F	CGAGCCCACCGGGAACGAAA	human
IL6-R	GGACCGAAGGCGTTGTGGAG	human
IL17A-F	AATCTCCACCGCAATGAGGA	human
IL17A-R	ACGTTCCCATCAGCGTTGA	human
IL17F-F	AACCAGCGCGTTTCCATGTCAC	human
IL17F-R	GAGCATTGATGCAGCCCAAGTTC	human
TNF-F	CGCCGTCTCCTACCAGACCAAGGTCAAC	human
TNF-R	ATGATCCCAAAGTAGACCTGCCCAGACTCG	human
CCL2-F	AGTCTCTGCCGCCCTTCT	human
CCL2-R	GTGACTGGGGCATTGATTG	human
RANKL-F	TGGATCACAGCACATCAGAGCAG	human
RANKL-R	TGGGGCTCAATCTATATCTCGAAC	human
OPG-F	GTGTGCGAATGCAAGGAAGG	human
OPG-R	CCACTCCAAATCCAGGAGGG	human
ALP-F	ACAAGCACTCCCACTTCATCTGGA	human
ALP-R	TCACGTTGTTCCTGTTCAGCTCGT	human
PTH1R-F	TCACCGTAGCTGTGCTCATCCT	human
PTH1R-R	GAGTAGAGCACAGCGTCCTTGA	human
COL1A1-F	CTCCTGACGCACGGCC	human
COL1A1-R	CCGTTCTGTACGCAGGTGATT	human
COL17A1-F	GCTCTTGGCATTCCTAGTGGTC	human
COL17A1-R	GATGTACTGCTGAATCTCCTGGC	human
GAPDH-F	AGGTCATCCCAGAGCTGAACG	mouse
GAPDH-R	CACCCTGTTGCTGTAGCCGTAT	mouse
OC-STAMP-F	ATGAGGACCATCAGGGCAGCCACG	mouse
OC-STAMP-R	GGAGAAGCTGGGTCAGTAGTTCGT	mouse
DC-STAMP-F	ACTAGAGGAGAAGTCCTGGGAGTC	mouse
DC-STAMP-R	CACCCACATGTAGAGATAGGTCAG	mouse
NFATC1-F	CCCGTCACATTCTGGTCCAT	mouse
NFATC1-F	CAAGTAACCGTGTAGCTGCACAA	mouse
RANK-F	TTTGTGGAATTGGGTCAATGAT	mouse
RANK-R	ACCTCGCTGACCAGTGTGAA	mouse
CTSK-F	AGGCAGCTAAATGCAGAGGGTACA	mouse
CTSK-R	AGCTTGCATCGATGGACACAGAGA	mouse
CALCR-F	CGCATCCGCTTGAATGTG	mouse
CALCR -R	TCTGTCTTTCCCCAGGAAATGA	mouse
IL17A-F	GCCCTCAGACTACCTCAACC	mouse
IL17A-R	GTCCTAGTAGGGAGGTGTGAAGTTG	mouse
IL17F-F	AATTCCAGAACCGCTCCAGT	mouse
IL17F-R	TTGATGCAGCCTGAGTGTCT	mouse
TNF-F	TCTGTCTACTGAACTTCGGGGTG	mouse
TNF-R	ACTTGGTGGTTTGCTACGACG	mouse
CCL2-F	TCTCCAGCCTACTCATTGGG	mouse
CCL2-R	AGGTCCCTGTCATGCTTCTG	mouse
